# Exploring the Link between Micronutrients and Phytoplankton in the Southern Ocean during the 2007 Austral Summer

**DOI:** 10.3389/fmicb.2012.00202

**Published:** 2012-07-10

**Authors:** Christel S. Hassler, Marie Sinoir, Lesley A. Clementson, Edward C. V. Butler

**Affiliations:** ^1^Plant Functional Biology and Climate Change Cluster, University of Technology SydneyBroadway, NSW, Australia; ^2^Marine and Atmospheric Research, Commonwealth Scientific and Industrial Research OrganisationHobart, TAS, Australia; ^3^University of TasmaniaSandy Bay, TAS, Australia; ^4^Ultramarine ConceptsSandy Bay, TAS, Australia

**Keywords:** subantarctic zone, pigments, Zn, Co, SAZ-Sense, trace element, subantarctic, polar

## Abstract

Bottle assays and large-scale fertilization experiments have demonstrated that, in the Southern Ocean, iron often controls the biomass and the biodiversity of primary producers. To grow, phytoplankton need numerous other trace metals (micronutrients) required for the activity of key enzymes and other intracellular functions. However, little is known of the potential these other trace elements have to limit the growth of phytoplankton in the Southern Ocean. This study, investigates whether micronutrients other than iron (Zn, Co, Cu, Cd, Ni) need to be considered as parameters for controlling the phytoplankton growth from the Australian Subantarctic to the Polar Frontal Zones during the austral summer 2007. Analysis of nutrient disappearance ratios, suggested differential zones in phytoplankton growth control in the study region with a most intense phytoplankton growth limitation between 49 and 50°S. Comparison of micronutrient disappearance ratios, metal distribution, and biomarker pigments used to identify dominating phytoplankton groups, demonstrated that a complex interaction between Fe, Zn, and Co might exist in the study region. Although iron remains the pivotal micronutrient for phytoplankton growth and community structure, Zn and Co are also important for the nutrition and the growth of most of the dominating phytoplankton groups in the Subantarctic Zone region. Understanding of the parameters controlling phytoplankton is paramount, as it affects the functioning of the Southern Ocean, its marine resources and ultimately the global carbon cycle.

## Introduction

The circumpolar Subantarctic Zone (SAZ) is an important biome of the global ocean which separates the High Nutrient Low Chlorophyll (HNLC) Southern Ocean from the mostly Low Nutrient Low Chlorophyll (LNLC) subtropical water from the Indian, Pacific, and Atlantic Oceans (Bowie et al., 2011a). The SAZ region forms a “belt” of important carbon sequestration accompanied by a phytoplankton biomass characterized by a low seasonality (Banse, 1996; Metzl et al., 1999; McNeil et al., 2001). However it is still unclear what parameters mostly constrain phytoplankton growth and how the dynamics of the SAZ region will be affected in the future.

The recent SAZ-Sense project took advantage of the natural variability in the SAZ region around Tasmania to study the impact of predicted future changes that will affect its functioning. This project focused on parameters likely to control phytoplankton biomass and carbon fixation, such as macro- (N, P, and Si) and micronutrients (essential trace metals), grazing and cell lysis promoted by the microbial loop, and as well, carbon export. Project outputs would help resolve the extent of future SAZ contribution to atmospheric CO_2_ fixation and climate regulation. The SAZ region west of Tasmania (W-SAZ) is characteristic of most of the SAZ region nowadays (Trull et al., 2001), whereas the region east of Tasmania (E-SAZ), with greater intrusion of macronutrient-poor, northern subtropical waters, and enhanced micronutrient inputs (e.g., iron, Hill et al., 2008; Bowie et al., 2009) mimics future predicted changes in the SAZ region. The two contrasting regions were compared to the waters south of Tasmania in the Polar Frontal Zone (PFZ) as an example of typical HNLC waters that could fuel the SAZ more significantly in the future (Herraiz Borreguero and Rintoul, 2011). The scientific rationale behind this study is summarized in Bowie et al. (2011a) and most of the results have been published in a special issue in Deep-Sea Research II (Table A1 in Appendix).

The difference in phytoplankton communities observed in the E-SAZ and the W-SAZ cannot be solely explained by considering macronutrients, light, grazing rates, and temperature (Kidston et al., 2011; Mongin et al., 2011; Pearce et al., 2011). It is believed that iron and silicic acid are mostly limiting the growth of phytoplankton, and thus, their ability to fix carbon (Bowie et al., 2009, 2011a; Lannuzel et al., 2011). Results from the SAZ-Sense project, demonstrate that silicic acid was limiting diatoms (de Salas et al., 2011; Fripiat et al., 2011) and iron was potentially limiting the growth of entire phytoplankton communities both in the W-SAZ and the PFZ (Lannuzel et al., 2011). Maximum photosynthetic quantum yield and results from iron phytoplankton uptake rates suggest that the W-SAZ was the main iron-limited region at the time (Schoemann et al., unpublished). In the E-SAZ, iron was not limiting (Lannuzel et al., 2011) and diatoms were practically absent. Analysis of the phytoplankton communities revealed marked differences among the W-SAZ, PFZ, and E-SAZ, with the E-SAZ being dominated by nanoplankton (2–20 μm) and dinoflagellates (de Salas et al., 2011; Pearce et al., 2011). In the E-SAZ, despite possible light limitation (euphotic depth of 47 m and shallow mixed layer depth (MLD) of 16 m, but main mixed layer down to 79 m; Mongin et al., 2011; Westwood et al., 2011; Table A1 in Appendix), the parameters controlling phytoplankton growth remain largely unresolved. Interestingly, phytoplankton carbon fixation and export were comparable or greater in the W-SAZ and the E-SAZ, despite a 1.4-fold average greater integrated chlorophyll *a* in the E-SAZ (Ebersbach et al., 2011; Jacquet et al., 2011; Westwood et al., 2011; Table A1 in Appendix), demonstrating that iron limitation and carbon fixation and export were not directly related during SAZ-Sense. If the E-SAZ, represents the future of the SAZ region, then iron limitation is likely to be decreased and other micronutrients could play a role in capping phytoplankton growth, particularly if they are supplied by pathways differing from iron. It, thus, becomes urgent to explore the potential of other micronutrients to co-limit the growth of phytoplankton.

This study investigates the role of other micronutrients Zn, Co, Cu, which share several properties with iron in limiting the growth of phytoplankton, or at the least, in mediating community structure (Morel et al., 1994; Sunda and Huntsman, 1995a,b; Buitenhuis et al., 2003; Saito and Goepfert, 2008; Saito et al., 2008). These micronutrients are co-factors of enzymes that catalyze essential reactions for the growth of phytoplankton, and key structural compounds (Morel and Price, 2003; Morel et al., 2003). Their possible limitation in marine systems are suggested by several laboratory studies (e.g., Morel et al., 1994; Saito et al., 2010) and phytoplankton have evolved high affinity uptake transport systems to survive under limited supply (Sunda and Huntsman, 1995b; Saito et al., 2002). These micronutrients are present at low concentrations in the open ocean (Lohan et al., 2002; Ellwood et al., 2005; Ellwood 2008; Saito et al., 2010; Croot et al., 2011; Butler et al., in revision), they readily associate with organic compounds, and thus, are mostly complexed by organic ligands (Moffett and Brand, 1996; Saito and Moffett, 2001; Ellwood, 2004, 2008; Ellwood et al., 2005; Lohan et al., 2005), which decrease their bioavailability to sustain phytoplankton growth. This study also includes other potentially interesting micronutrients recently reported as essential in marine phytoplankton (Cd, Ni, e.g., Sunda and Huntsman, 2000; Lane et al., 2005; Dupont et al., 2008, 2010).

Because different phytoplankton species and functional groups have different biological requirements for growth (Buitenhuis et al., 2003; Sarthou et al., 2005; Sedwick et al., 2007; Saito and Goepfert, 2008) leading to differences in intracellular nutrient quota and drawdown (de Baar et al., 1997; Arrigo et al., 1999; Ho et al., 2003; Quigg et al., 2003, 2011; Twining et al., 2004a,b; Finkel et al., 2007, 2010), we investigate the relation between the distributions of dissolved nutrients and phytoplankton biomarker pigments, which can be used to infer phytoplankton community composition by using CHEMTAX at 5 locations during the SAZ-Sense expedition (e.g., de Salas et al., 2011). In addition, biological requirement for micronutrients is related to the size-aspect ratio (Sarthou et al., 2005; Hassler and Schoemann, 2009; Finkel et al., 2010). Size fractionation of pigments also pointed to different dominating phytoplankton groups in the SAZ and the Tasman Sea (de Salas et al., 2011; Hassler et al., 2011; Pearce et al., 2011). For these reasons, the link between micronutrients with large and small phytoplankton biomarker pigments is studied to gain further insight into their potential control on phytoplankton biomass, biodiversity, and contribution to carbon export. As done in previous studies, the disappearance ratio of nutrients is discussed to gain an understanding of the underlying processes at play (e.g., de Baar et al., 1997; Arrigo et al., 1999; Saito et al., 2010; Croot et al., 2011).

## Materials and Methods

### Study region

**Figure 1:**
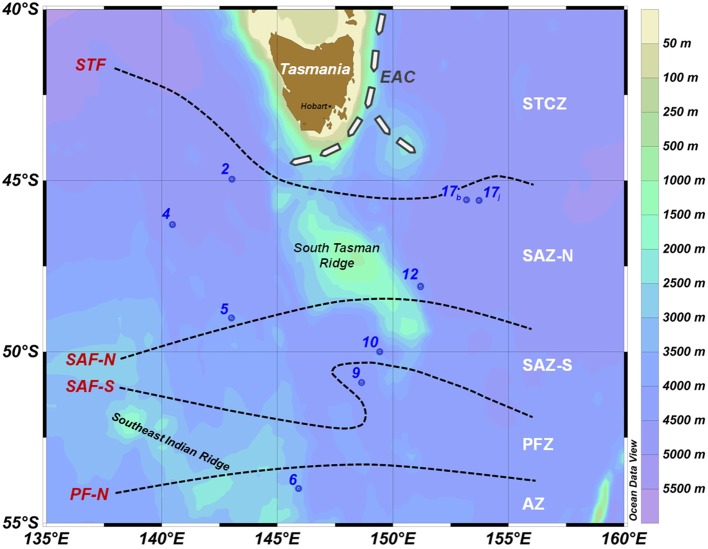
**Location of stations (number) as part of the SAZ – Sense voyage (January–February 2007) superimposed on bathymetry**. The notations to the left of the dashed lines indicate the approximate location of fronts (following Sokolov and Rintoul, 2002) at the time of this voyage. The notations to the right define the zones between the fronts. The dashed arrows, tagged with EAC, indicate the East Australian Current extension characterized by mesoscale eddy features; the path around the south of Tasmania is the Tasman Outflow (Ridgway, 2007). Fronts (red text on left): STF, Subtropical Front; SAF-N, Subantarctic Front–North; SAF-S, Subantarctic Front–South; PF-N, Polar Front–North. Zones between fronts (white text on right): STCZ, Subtropical Convergence Zone; SAZ-N, Subantarctic Zone–North; SAZ-S, Subantarctic Zone–South; PFZ, Polar Frontal Zone; AZ, Antarctic Zone. Station 17 was sampled twice (on cast b and j on the 11 February 2007 and 17 February 2007, respectively).

The SAZ-Sense voyage (*RV Aurora Australis*, 17 January–20 February 2007) visited contrasting water masses in the SAZ region around Tasmania (E-SAZ and W-SAZ) as well as further south in the PFZ, and crossed several fronts as summarized in Figure 1. The oceanography of the region is highly variable as a result of spatially heterogeneous inputs from subtropical waters from the north, and Subantarctic Mode Water and Antarctic Intermediate Water from the South (Bowie et al., 2011a; Herraiz Borreguero and Rintoul, 2011); nutrients from dust deposition and the continental margin (e.g., Bowie et al., 2009) provide a further overlay. The whole system creates naturally contrasting regions relevant to the study of controls on phytoplankton dynamics and their associated carbon fixation, recycling, and export. Oceanography of the region is summarized and put in a larger context elsewhere (Bowie et al., 2011b; Herraiz Borreguero and Rintoul, 2011). Critical properties of selected stations are summarized in Table A1 in Appendix.

The SAZ-Sense project took advantage of the natural variability in the SAZ region around Tasmania to study the impact of predicted changes in major currents (e.g., Antarctic Circumpolar Current (ACC), East Australian Current (EAC, Hill et al., 2008; Toggweiler and Russell, 2008) that modulate important parameters for phytoplankton growth, such as the depth of the mixed layer, and micro- and macronutrient input (Bowie et al., 2009). The SAZ region west of Tasmania (W-SAZ) has low phytoplankton biomass characteristic of much of the SAZ region as it is nowadays (Trull et al., 2001), whereas the region East of Tasmania (E-SAZ) is characterized by high biomass persisting from the spring to summer, likely due to micronutrient (iron) input from eddy fields, arising from EAC activity and from Australian dust (Bowie et al., 2009, 2011a; Lannuzel et al., 2011; Mongin et al., 2011). In the future, the EAC is expected to strengthen (Hill et al., 2008) possibly bringing more eddies into the E-SAZ and Australian continent will become drier, with more terrigenous input from dust storms and bushfires expected to deposit in the South Tasman Sea (Matear et al., submitted).

### Water sampling

Samples for trace metal analyses and some pigment analyses (Stns 2, 6, 17, Figure 1) were taken using Teflon-coated Niskin X-1010 bottles (General Oceanics, USA) mounted on an autonomous rosette (Model 1018, General Oceanics, USA) and deployed using a Kevlar hydroline (Strongrope, AU). Water samples for micronutrient analysis were collected and filtered (Pall, Acropack 200) in a clean van under a HEPA filter (ISO Class 5 conditions). Sample acquisition and handling was as per GEOTRACES recommendations^1^, using acid-washed non-contaminating material as detailed elsewhere (Bowie et al., 2009; Lannuzel et al., 2011). Samples were acidified using quartz-distilled HCl (1 mL L^−1^, Seastar Baseline, Canada) and kept for 6 months doubly bagged in plastic boxes in clean storage, prior to analysis.

Salinity, temperature, fluorescence, and oxygen were obtained from calibrated conductivity-temperature-depth (CTD, SeaBird SBE9plus) data using water collected from Niskin bottles (General Oceanics) per Rosenberg (2007). Macronutrients (reactive phosphorus, PO_4_; silicic acid, Si; nitrate-plus-nitrite, NO_x_; ammonium, NH_4_) were obtained by the analysis of unfiltered water using automated flow-injection analyzer and colorimetric techniques (Watson et al., 2005; Rosenberg, 2007). Samples were also collected for pigment determination (Stns 4, 9, 12). CTD deployments were performed close (within 0.1° latitude and longitude and 3.2 h) to the autonomous rosette to ensure that profiles of trace elements and other parameters describe the same water column (Bowie et al., 2011a).

### Micronutrients

Micronutrients were determined in an ISO class 5 Clean laboratory using automated flow-injection, solid phase extraction coupled to inductively coupled plasma-mass spectrometry (FI-SPE-ICP-MS; method and performance described in Butler et al., in revision, and full details in O’Sullivan et al. in preparation; Table 1). Briefly, 7.0-mL subsamples are decanted into acid-cleaned, 10-mL screw-cap polypropylene tubes, which are set in the tray of a CETAC ASX-520 auto sampler. Initiation of the ICP-MS control program sees the preconcentration/matrix-elimination step proceed on a PerkinElmer FIAS 400 system, with the sample stream merged with cleaned 1 M ammonium acetate buffer. The buffered sample (pH 5.7 ± 0.2) is then passed through the iminodiacetate (IDA) chelating sorbent (Toyopearl AF chelate-650M resin, 40–90 μm, Tosoh Bioscience GmbH, Germany) packed in 1-cm Global FIA (USA) cartridge mounted in the switching valve position. After loading, the solid phase is rinsed with buffered Milli-Q (Millipore, USA) deionized water to flush. With the switching valve moved to “Inject,” the eluent [0.8 M HNO_3_ (Seastar, Canada) +internal standard 10 ppb Rh] carries the adsorbed trace metals directly into the nebuliser (concentric quartz Meinhard-type with cyclonic spray chamber) and then into the quadrupole ICP-MS (PerkinElmer Elan DRC II, USA). The instrumental conditions for ICP-MS operation are summarized in Table 1. With each analysis of two casts (24 samples), calibration standards (standard additions to seawater sample for matrix matching), blanks and reference seawaters (NASS 5, SAFe S, and D2) were run. The instrument was controlled, and data collected and put through primary processing, using Elan v3.4 software, with subsequent data processing done with MS Excel. “Figures of Merit” for this analytical method are presented in Table 2. Based on accuracy, blank, and performance in the measurement of reference seawaters (NASS 5, SAFe S, and D2), this version of the technique can be used for the measurement of Zn, Co, Cd, Cu, Ni, and Pb in seawater.

**Table 1:** **FI–SPE–ICP-MS conditions for determination of trace metals in seawater (O’Sullivan et al., in preparation)**.

Typical ICP-MS Conditions	
Nebuliser gas flow (mL/min)	1.02
Auxiliary gas flow (mL/min)	1.2
Plasma gas flow (mL/min)	15
ICP RF power (W)	1400
Skimmer/sample cones	Platinum
Sweep/readings	1
Readings/replicates	60
Number of replicates	1
Process signal profile	Sum
Dwell time (ms)	40
No of isotopes analyzed	21
Integration time (ms)	2400

**Table 2:** **Figures of Merit for FI–SPE–ICP-MS analyses**.

	Detection limit^a^ and precision ^b^	SAFe S	SAFe D2	NASS 5
^59^Co	0.003	0.006 ± 0.002	0.032 ± 0.003	0.188 ± 0.012
	8%	(0.005 ± 0.002)	(0.045 ± 0.004)	(0.187 ± 0.05)
^60^Ni	0.03	2.47 ± 0.19	8.84 ± 0.51	4.52 ± 0.31
	6%	(2.31 ± 0.10)	(8.58 ± 0.30)	(4.31 ± 0.48)
^65^Cu	0.05	0.56 ± 0.07	2.26 ± 0.11	4.82 ± 0.25
	5%	(0.51 ± 0.05)	(2.25 ± 0.11)	(4.67 ± 0.72)
^66^Zn	0.22	<0.22	7.09 ± 0.49	1.42 ± 0.08
	7%	(0.064 ± 0.019)	(7.20 ± 0.50)	(1.56 ± 0.60)
^111^Cd	0.005	<0.005	0.84 ± 0.02	0.178 ± 0.015
	3%	(0.001 ± 0.0002)	(0.986 ± 0.027)	(0.205 ± 0.030)

*Average metal concentration (±1 standard deviation) are given for SAFe S 430 and 125 (*n* = 10), SAFe D2 414 (*n* = 8), and NASS 5 (*n* = 8) reference samples. Consensus and certified values for these samples are given in bracket (http://www.es.ucsc.edu/~kbruland/GeotracesSaFe/kwbGeotracesSaFe.html, Consensus values from November 2011). Concentrations are given in nmol/kg. Note that here no UV-irradiation is done prior to analysis*.

*^a^Detection limit calculated on 3 × standard deviation of 0.1% HCl blank (*n* = 8)*.

*^b^Coefficient of variation of SAFe seawater D2 (*n* = 8)*.

Seawater samples were not UV irradiated before analysis. Very recent work indicates that not all dissolved species of Co, and possibly Cu, in seawater will be measured under such circumstances, because some strongly complexed forms of these two metals only become detectable after photo-oxidative destruction of the organic ligands (Shelley et al., 2010; Biller and Bruland, 2012). Our method detects dissolved *labile* concentrations of metals; those which can be displaced by the IDA chelating sorbent. For the other metals Cd, Ni, Zn, and Pb, dissolved labile concentrations are comparable to total dissolved concentrations in oceanic waters. For our purposes, dissolved labile concentrations determined directly by the FI-SPE-ICP-MS method are a useful representation of the bioavailable fraction.

### Pigment analysis

Pigment samples were collected in two size fractions, [≥10 μm (L-phyto) and 0.8–10 μm (S-phyto)] using gentle sequential filtration (<5 mmHg) of approximately 1 L of seawater through 10 and 0.8-μm polycarbonate filters (Millipore). Filters were then stored in cryo-vials in liquid nitrogen prior to being analyzed back on shore. To extract the pigments, the filters were cut into small pieces and covered with 100% methanol (3 mL) in a 10-mL centrifuge tube. The samples were vortexed for about 30 s and then sonicated in an ice-water bath for 15 min in the dark. The samples were then kept in the dark at 4°C for approximately 15 h. After this time, 200 μL water was added to the methanol such that the extract mixture was 90:10 methanol:water (vol:vol) and sonicated once more in an ice-water bath for 15 min. The extracts were quantitatively transferred to a clean a centrifuge tube and centrifuged to remove the filter paper. The final extract was filtered through a 0.2-μm membrane filter (Whatman, Anatop) prior to analysis by HPLC using a Waters – Alliance high performance liquid chromatography system, comprising a 2695XE separations module with column heater and refrigerated auto sampler and a 2996 photo-diode array detector. Immediately prior to injection the sample extract was mixed with a buffer solution (90:10 28 mM tetrabutyl ammonium acetate, pH 6.5: methanol) within the sample loop. After injection pigments were separated using a Zorbax Eclipse XDB-C8 stainless steel 150 mm × 4.6 mm ID column with 3.5 μm particle size (Agilent Technologies) and a binary gradient system with an elevated column temperature following a modified version of the van Heukelem and Thomas (2001) method. The separated pigments were detected at 436 nm and identified against standard spectra using Waters Empower software. Concentrations of chlorophyll *a* (Chl*a*), chlorophyll *b* (Chl*b*), and β,β-carotene in sample chromatograms were determined from standards (Sigma), while all other pigment concentrations were determined from standards (DHI, Denmark).

Pigments which relate specifically to an algal class are termed marker or diagnostic pigments (Jeffrey and Vesk, 1997; Jeffrey and Wright, 2006). Some of these diagnostic pigments are found exclusively in one algal class (e.g., alloxanthin in cryptophytes), while others are the principal pigments of one class, but are also found in other classes (e.g., fucoxanthin in diatoms and some haptophytes; 19′-butanoyloxyfucoxanthin in chrysophytes and some haptophytes). The presence or absence of these diagnostic pigments can provide a simple guide to the composition of a microalgal community, including identifying classes of small flagellates that cannot be determined by light microscopy techniques. Given that our dataset is limited, it was validated by comparison to the extensive total Chl*a* measurements made by de Salas et al. (2011) and relative fluorescence recorded from the CTD. To push the comparison further, the CHEMTAX matrix used in de Salas et al. (2011) was applied to our data from stations 2, 6, and 17 to represent dominating large and small phytoplankton groups in the W-SAZ, PFZ, and E-SAZ, respectively.

### Data analyses and plots

Sections of the voyage track (Figure 1) were plotted using Ocean Data View (version 4.5; Schlitzer, 2012). Since our goal is to compare micronutrients’ distribution with phytoplankton biomass, size class, and functional groups, data above 125 m depth are presented. Full depth profiles and oceanography of trace elements have been discussed elsewhere (Lannuzel et al., 2011; Butler et al., in revision). The disappearance ratio between micronutrients with macronutrients (e.g., dCo/dPO_4_, Arrigo et al., 1999, also referred to the dissolved ecological stoichiometric ratio, Saito et al., 2010) was used as a proxy for phytoplankton uptake and growth rate (Arrigo et al., 1999; Cullen et al., 2003; Finkel et al., 2010; Croot et al., 2011). To identify the potential of each micronutrient to limit the growth of phytoplankton, the disappearance ratio is compared to phytoplankton cellular quotas, reflecting their biological requirement for growth, and dissolved macronutrient (e.g., NO_x_/PO_4_) and micronutrient (e.g., dZn/dCd) spot ratios, reflecting maximal micronutrient stock available for phytoplankton growth. It is to be noted that chemical speciation is not considered in this study. The labile dissolved metal considered likely overestimates the fraction that is bioavailable to support phytoplankton growth.

The statistical relations between micro- and macronutrient with temperature, salinity, chlorophyll *a*, and biomarker pigments were analyzed using the Excel package XLSTAT. Depths of the mixed layer and the euphotic zone (Westwood et al., 2011) were used to define the depths considered in statistical tests and comparison (0–100 m for stations 9, 0–75 m for other stations; Table A1 in Appendix). This results in dataset of 32 observations for which measurements of physical parameters, micro- and macronutrients, as well as biological parameters, were considered. Correlation tests were done using Pearson’s coefficient with a 95% interval of confidence to highlight the occurrence of significant positive or negative linear relationship between two parameters. The most significant relationships are graphically presented. Canonical Correspondence Analysis (CCA) was done to extract the pattern and illustrate the relation existing between the different parameters and observations. CCA was applied to represent the correspondence between (i) biological and physical parameters with the chemistry of the sites, and (ii) small and large phytoplankton communities and micronutrients.

## Results

### Water masses

**Figure 2:**
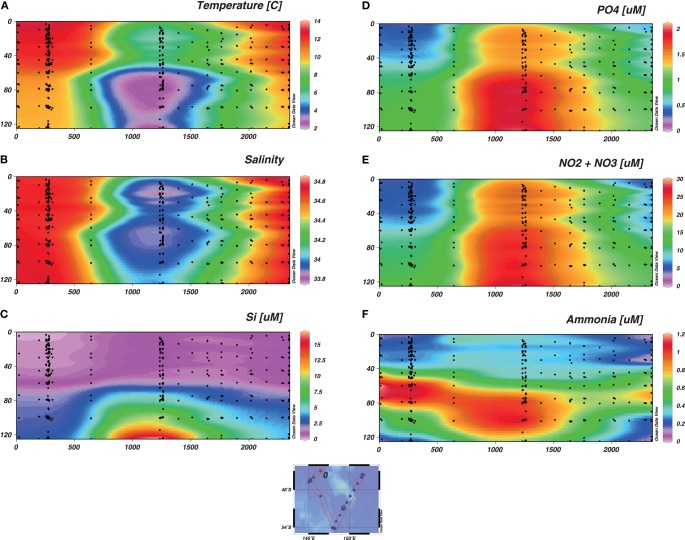
**Parameters collected from the regular CTD cast represented as a function of depth**. The section represents the whole voyage track from Stn 2 to Stn 17, with the W-SAZ (Stn 4) being the second station on the left, the PFZ (Stn 6) in the center and the E-SAZ (Stn 17) the last on the right. The temperature **(A)**, salinity **(B)** and macronutrients [Si **(C)**; PO_4_, **(D)**; NO_x_, **(E)**; and ammonia, **(F)**] are shown.

A snapshot of the hydrology along the relevant portion of the SAZ-Sense voyage track is shown in Figure 2. Temperature and salinity (Figures 2A,B, respectively) broadly characterize the regional water masses (see also Bowie et al., 2011b, with front definitions after Sokolov and Rintoul, 2002). The bulk was SAZ with salinity and temperature in the respective ranges, 34.2–34.8 and 2–12°C. The northernmost stations, west and east, were very close to the Subtropical Front (STF) with salinities at 34.9 (and temperature at 150 falling from 12 to 10°C across the STF, data not shown). The Polar Front is classically delineated by the subsurface *T*_min_ < 2°C, that occurs deeper than the section shown. A less precise indicator is surface salinity falling below 34 for the southernmost station (Figure 2B). The oceanography of the region during the SAZ-Sense expedition is fully described in Bowie et al. (2011b).

### Macronutrients

Sections of Si, PO_4_, NO_x_, and NH_4_ are depicted in Figures 2C–F, respectively. PO_4_ and NO_x_ showed very similar progressions along the voyage track. In northern SAZ surface waters, PO_4_ was drawn down to ≤0.5 μM and NO_x_ to ≤5 μM. Beyond the SAF-S front, concentrations began to ramp up, with surface waters ~1.5 μM PO_4_ and ~20 μM NO_x_. The nutricline typically varied between 60 and 80 m throughout the study region. Si was strongly depleted to a few micromolar concentrations in the upper 60 m for all stations (Figure 2C). Only beneath the nutricline, and poleward of SAF-S into PFZ waters, did Si begin to increase in concentration. NH_4_ ranged from undetectable (<0.01 μM) to <0.4 μM in surface waters (Figure 2F). It has distinct subsurface maxima at the nutricline and deeper (to 120 m) at all stations, but they are more pronounced (>1 μM) on the western side of the voyage track than the east. Beyond 200 m (data not shown), NH_4_ declined rapidly to background levels (≤0.01 μM). NH_4_ is a useful indicator of remineralization of organic matter. Data demonstrated a significant remineralization just below the MLD in the W-SAZ and in the PFZ but not in the E-SAZ (Figure 2; Table A1 in Appendix).

### Micronutrients

**Figure 3:**
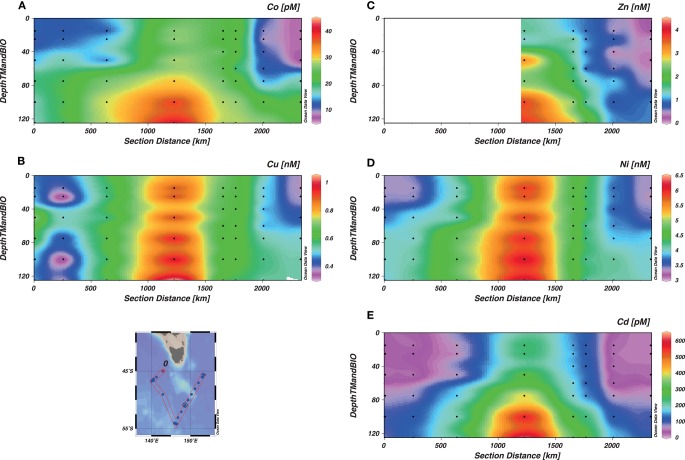
**Concentration of trace elements as a function of depth**. The section represents the whole voyage track from Stn 2 to Stn 17, with the W-SAZ (Stn 4) being the second station on the left, the PFZ (Stn 6) in the center and the E-SAZ (Stn 17) the last on the right. The micronutrients Co **(A)**, Cu **(B)**, Zn **(C)**, Ni **(D)**, and Cd **(E)** are shown.

Dissolved labile concentrations of micronutrient metals along the voyage track are shown in Figure 3. Complete records have been plotted for Cd, Co, Cu, and Ni, but stations before Stn 6 were assessed as contaminated for Zn. The contaminated results were identified by irregularities in the depth profiles and recurrent outliers in property-property plots (i.e., metal vs. hydrological properties, metal vs. macronutrient (e.g., Si), and metal vs. metal). The distributions of these micronutrients in the SAZ-Sense study region is fully described in Butler et al., in revision. Cd, Cu, and Ni were closely related to PO_4_ (Figure 2D and below), but the extent of their biological depletion was quite different. Relative to a reference depth of 150–200 m (see Bowie et al., 2011b), the depletion of Cd was 6 to >96%, that of Cu 11–35%, and Ni 7–20%. For each, the surface water depletion declined poleward. Co was also strongly depleted (31–70%). Zn was more comparable to the macronutrient Si in being depleted in most surface waters. Its depletion often extended well into the water column (≥200 m), so a reference depth of 150–200 m was not suitable for evaluating degree of depletion.

### Nutrients spot and disappearance ratio

**Figure 4:**
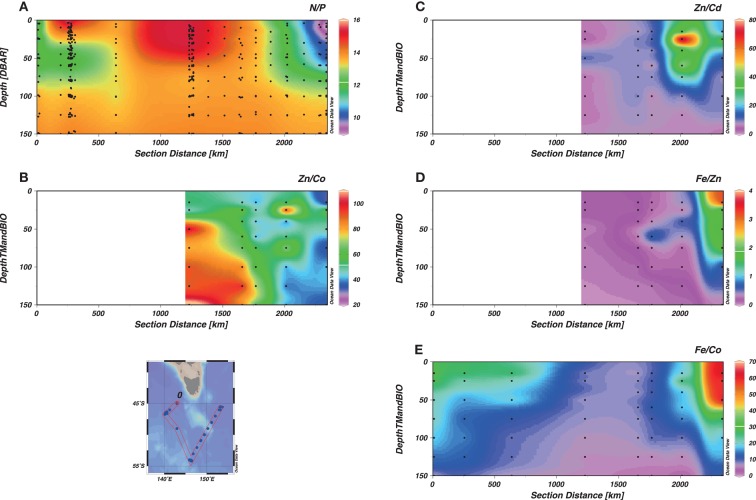
**Ratio of macro- and micro-nutrients dissolved concentrations as a function of depth**. The section represents the whole voyage track from Stn 2 to Stn 17, with the W-SAZ (Stn 4) being the second station on the left, the PFZ (Stn 6) in the center and the E-SAZ (Stn 17) the last on the right. NO_x_/PO_4_ [N/P **(A)**], dZn/dCd [Zn/Cd **(B)**], dZn/dCo [Zn/Co **(C)**], dFe/dZn [Fe/Zn **(D)**], and dFe/dCo [Fe/Co **(E)**] are shown. Data for Fe are from Lannuzel et al. (2011).

**Figure 5:**
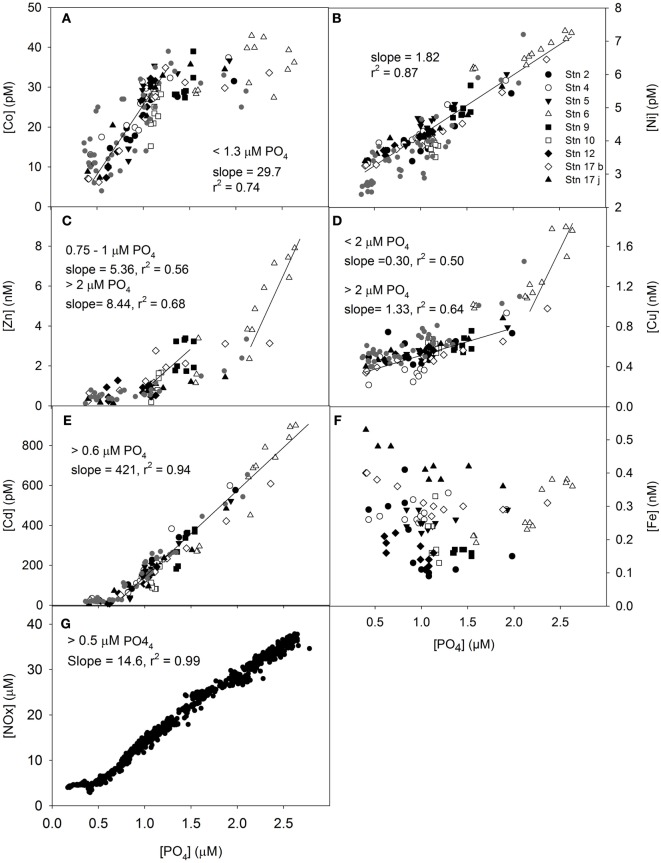
**Relationship between micronutrient and phosphate dissolved concentrations up to 1000 m depth for Co (A), Zn (B), Cd (C), Ni (D), Cu (E), Fe (F), and NO_x_ (G)**. Significant linear relationships are shown with their coefficient of correlation and slope. Stations are represented by different symbols. Data from a previous study in the region are also shown (Ellwood, 2008). Data for dissolved iron are from Lannuzel et al. (2011).

**Figure A1:**
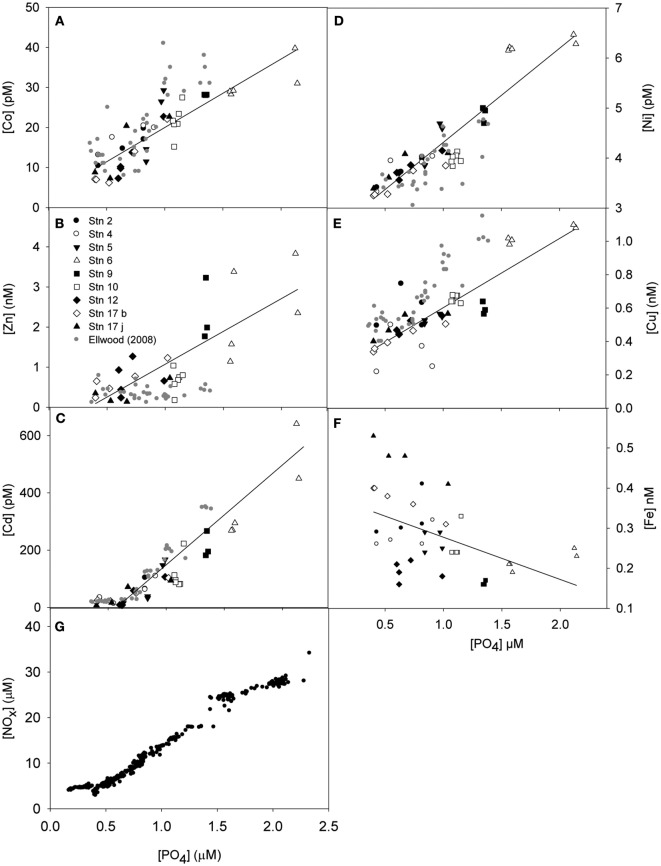
**Disappearance ratio of dissolved micronutrients Co (A), Zn (B), Cd (C), Ni (D), Cu (E), Fe (F) and macronutrient (NO_x_, G) with dissolved phosphate (PO_4_) for the top 100 m for the Subantarctic and Polar Frontal Zone (Australian sector)**. Depths and stations are distinguished by different symbol. The slope and the coefficient of correlation for linear regression are shown. Data from a previous study in the region is also shown (Ellwood, 2008). It is to be noted that Ellwood (2008) did not cross the North Polar Front into Antarctic Zone, which could explain some discrepancy. Data for Fe are from Lannuzel et al. (2011).

Considering data from all depths, the macronutrient ratios between NO_x_ and PO_4_ for the study region were close to an optimal value of 16 [14.6 ± 0.07 (standard error), *r*^2^ = 0.98, *n* > 750, Figure 5G; Redfield et al., 1963]. However, a kink was observed for [PO_4_] < 0.5 μM (Figure 5G), suggesting that less NO_x_ was relatively consumed in surface water of the W-SAZ and E-SAZ. The dissolved NO_x_/PO_4_ spot ratios are suboptimal in the W-SAZ (at depth) and the E-SAZ regions (Figure 4A). Considering data from 0 to 100 m depth only (Figure A1G in Appendix), a close to optimal disappearance ratio was observed (slope 18.0 ± 0.18, *r*^2^ = 0.98) for phosphate concentrations between 0.5 and 1.6 μM. For [PO_4_] > 1.6 μM, representing waters from the PFZ (Stn6), a significantly lower N/P disappearance ratio was observed (slope 7.00 ± 0.40, *r*^2^ = 0.85). Because Fe, Zn, and Co could limit or co-limit the growth of oceanic phytoplankton (e.g., Martin et al., 1990; Morel et al., 1994; Coale et al., 2003), and Zn, Co, Cd are interchangeable enzymatic co-factors that can all support the growth of some marine phytoplankton (Price and Morel, 1990; Sunda and Huntsman, 1995b; Timmermans et al., 2001), the ratios between these micronutrients were investigated (Figures 4B–E; Table A2 in Appendix). Dissolved zinc was present in excess compared to Co (dZn/dCo > 1, Figure 4B) with greatest ratios in the PFZ (Stn 6) and lowest ratios in the surface of the E-SAZ (Stn 17). The ratio dZn/dCd was close to unity in the PFZ and greater at Stn 12 just south of the E-SAZ. The ratios dFe/dZn and dFe/dCo (Figures 4D,E) were lower at the PFZ and greater in the surface waters of the E-SAZ. For all micronutrients, except Fe, a disappearance ratio could be calculated from the linear regression between dissolved micronutrient and PO_4_ concentrations (Table 3; Figure 5; Figure A1 in Appendix). A kink was observed at low PO_4_ concentrations (<0.5–0.6 μM PO_4_) for Cd and Zn and below 2 μM PO_4_ for Cu. Ni is the only micronutrient for which no kink was observed. For Zn, data suggested two kinks separated by a plateau between 1.5 and 2 μM PO_4_, however, the coefficient of correlation considering all data between 0 and 2 μM PO_4_ was only slightly lower (*r*^2^ = 0.47, slope 1764 μmol Zn mol^−1^ PO_4_, Figure 5B). For Co, Zn, and Cu inter-station differences were observed (Figure 5). For Co, Stns 6, and 9 were on the plateau (Figure 5A) and the slope dCo/PO_4_ was the greatest for Stns 5 and 10 (slope of 66–77 μmol mol^−1^) as compared to the other stations (32–49). For Zn, no correlation (*r*^2^ < 0.5) was observed for Stns 12 and 17, and greater dZn/PO_4_ slopes (9–11 μmol mol^−1^) were observed at Stns 9 and 10, suggesting greater Zn biological uptake at these stations. Correlations between Cu and PO_4_ dissolved concentrations (*r*^2^ > 0.5) were only observed at Stns 4 (*r*^2^ = 0.94, slope = 0.65), 5 (*r*^2^ = 0.78, slope = 0.26 mmol mol^−1^), 12 (*r*^2^ = 0.83, slope = 0.24 mmol mol^−1^), and 17 (*r*^2^ = 0.94 and 0.90, slope = 0.19 and 0.28 mmol mol^−1^). When considering only the data from the top 100 m for all stations, the relation between micronutrients and PO_4_ was slightly altered (Table 3; Figure A1, Table A2 in Appendix). In this case, the disappearance ratios followed the order Ni ~ Zn > Cu ~ Cd > Co. The disappearance ratio for Zn was 5.1 and 96 times greater than for Cd and Co, respectively, suggesting greater biological utilization of Zn as compared to Cd and Co. Similar ratios were obtained using NO_x_, whereas the significance of the correlation was much lower using Si (see Figure A1 in Appendix). Considering the disappearance ratios calculated in surface water (0–100 m) and at depth (125–1000 m) for each station (Table A1 in Appendix), revealed interesting trends. For all metals, except Co, the surface disappearance ratio in surface waters was equal or lower that the ratio calculated at depth. In addition, a loss of significant correlation between dissolved metal and phosphate was observed in the PFZ. Generally, a region between 49 and 50°S with greater disappearance ratios separated the surface waters between the SAZ and the PFZ (Table A1 in Appendix).

**Table 3:** **Summary of dissolved disappearance ratios and biological requirement for phytoplankton growth for Cd, Zn, Co, Ni, and Cu**.

Micronutrient (M)	dM/PO_4_	*r*^2^	Depth (m)	Location
**DISSOLVED DISAPPEARANCE RATIO (dM/PO_4_, μmol mol^−1^)**				
Cd	323	0.88	15–100	Southern Ocean (SAZ and PFZ, >0.6 μM PO_4_)^a^
	461	0.94	30–100	Southern Ocean (SAZ and PFZ, >0.6 μM PO_4_)^b^
	608	0.89	0–125	Southern Ocean (Ross Sea)^c^
	450	0.87	20–150	Subarctic Pacific (<1.2 μM PO_4_)^d,e^
Zn	1637	0.61	15–100	Southern Ocean (SAZ and PFZ, >0.6 μM PO_4_)^a^
	NA	0.001	30–100	Southern Ocean (SAZ and PFZ)^b^
	484	0.79	0–125	Southern Ocean (Ross Sea)^c^
	4857	0.73	>500	Southern Ocean (Drake passage and Zero meridian)^f^
	~2000–7000		0–400	
	251–370	0.70–0.99	8–150	Subarctic Pacific (<1.2 μM PO_4_)^d,g^
	797	0.99	30–300	Southern Ocean (Drake Passage)^g,h^
Co	17	0.79	15–100	Southern Ocean (SAZ and PFZ)^a^
	25	0.62	30–100	Southern Ocean (SAZ and PFZ)^b^
	19	0.54	0–125	Southern Ocean (Ross Sea)^c^
	35–40	0.98–0.99	8–150	Subarctic Pacific^d,g^
	38	0.87	5–500	Southern Ocean (Ross Sea)^h^
Ni	1881	0.84	15–100	Southern Ocean (SAZ and PFZ)^a^
	1903	0.72	30–100	Southern Ocean (SAZ and PFZ)^b^
	1181	0.61	0–125	Southern Ocean (Ross Sea)^c^
Cu	441	0.73	15–100	Southern Ocean (SAZ and PFZ)^a^
	598	0.80	30–100	Southern Ocean (SAZ and PFZ)^b^
	846	0.72	0–125	Southern Ocean (Ross Sea)^c^
	430–450	0.96–0.99	0–985	North Pacific^d,j,k^
	680	0.92	30–300	Southern Ocean (Drake Passage)^h,k^
**Strain**	Zn/P*^g^	Co/P**^g^	**Cu/P^k^**	
**BIOLOGICAL REQUIREMENT FOR PHYTOPLANKTON GROWTH (CELLULAR M/P, μmol mol^−1^)**				
*E. huxleyi*	1272 (0.7μ_max_)	264 (0.9μ_max_)	35 (0.9μ_max_, low Cu)	
			758 (0.9μ_max_, high Cu)	
*T. oceanica*	63 (0.9 μ_max_)	1314 (0.7μ_max_)	216 (0.9μ_max_)	
*T. pseudonana*	232 (0.6μ_max_)	1675 (0.5μ_max_)	NA	
*Synechococcus* sp	ND	8.5 (0.6μ_max_)	NA	

*^a^This study; ^b^Ellwood (2008); ^c^Fitzwater et al. (2000); ^d^Martin et al. (1989); ^e^Sunda and Huntsman (2000); ^f^Croot et al. (2011); ^g^Sunda and Huntsman (1995a); ^h^Martin et al. (1990), ^i^Saito et al. (2010); ^j^Bruland (1980); ^k^Sunda and Huntsman (1995b)*.

* No Co added, ** No Zn added. ND = not detected, NA = not applicable.

### Chlorophyll and biomarker pigments

**Figure 6:**
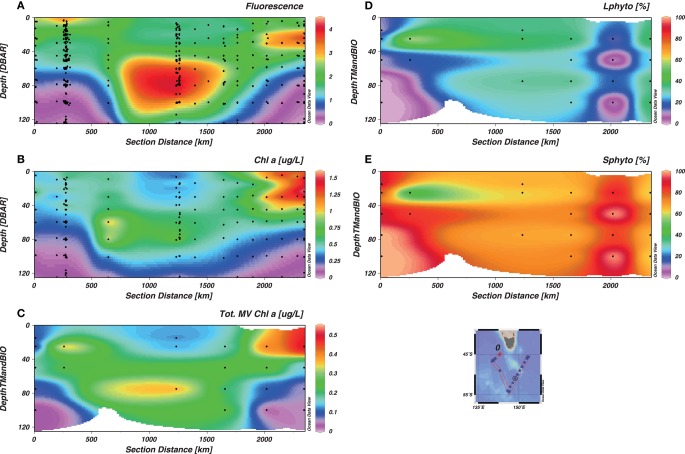
**Distribution of phytoplankton biomass as a function of depth**. The section represents the whole voyage track from Stn 2 to Stn 17, with the W-SAZ (Stn 4) being the second station on the left, the PFZ (Stn 6) in the center and the E-SAZ (Stn 17) the last on the right. The fluorescence from the CTD [CTDFLUORO **(A)**], the total chlorophyll *a* (Chl*a*) measured by the team of Simon Wright [de Salas et al., 2011 **(B)**], the total Chl*a* data used in this study **(C)** are shown. In addition, the relative size distribution between large [L-phyto, >10 μm **(D)**] and small phytoplankton [S-phyto, 0.8–10 μm, **(E)]** are shown.

Our dataset for total Chl*a* (Figure 6C) showed good correlation with the more extensive dataset from the fluorometer (CTD data, Figure 6A) and previous pigment analysis (de Salas et al., 2011, Figure 6B). The maximum Chl*a* concentration was observed in the surface of the E-SAZ (Stn 17) and the W-SAZ (Stn 4) and at depth in the PFZ (Stn 12). Total Chl*a* data from de Salas et al. (2011) were greater than our data, likely reflecting different extraction procedures and our sequential filtration step. Sequential filtration removes larger phytoplankton, decreasing clogging of the filter, and avoiding an overestimation of pigments’ concentration.

In the study region, contrasting distribution of phytoplankton from different size classes was observed (Figures 6D,E). Small phytoplankton (0.8–10 μm, S-phyto) dominated at all stations, except in surface water of the W-SAZ (stn 4). In the northern W-SAZ waters (Stn 2) and south of the E-SAZ (Stn 12), S-phyto represented >80% of the total Chl*a*. Large phytoplankton (≥10 μm, L-phyto) represented a significant fraction (25–40%) of total Chl*a* only in the PFZ, Stn 9, and in the E-SAZ.

**Figure 7:**
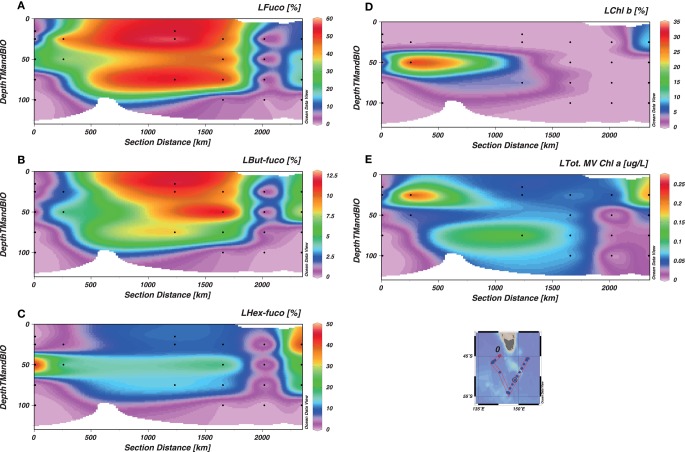
**Distribution of large phytoplankton (L, >10 μm) biomarker pigments as a function of depth**. The section represents the whole voyage track from Stn 2 to Stn 17, with the W-SAZ (Stn 4) being the second station on the left, the PFZ (Stn 6) in the center and the E-SAZ (Stn 17) the last on the right. Fucoxanthin [Fuco **(A)**], 19-But-fucoxanthin [But-Fuco **(B)**], 19 Hex-fucoxanthin [Hex-Fuco **(C)**], Chlorophyll *b* [Chl*b* **(D)**], and Total Chlorophyll *a* [Chl*a* **(E)**] are shown.

**Figure 8:**
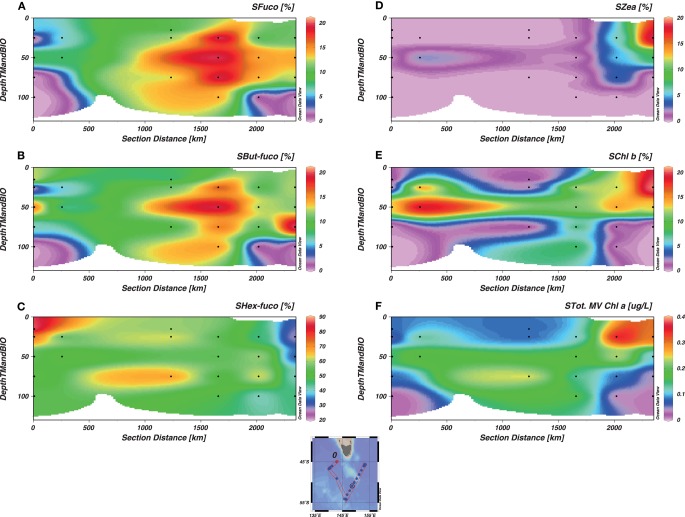
**Distribution of small phytoplankton (S, 0.8–10 μm) biomarker pigments as a function of depth**. The section represents the whole voyage track from Stn 2 to Stn 17, with the W-SAZ (Stn 4) being the second station on the left, the PFZ (Stn 6) in the center and the E-SAZ (Stn 17) the last on the right. Fucoxanthin [Fuco **(A)**], 19-But-fucoxanthin [But-Fuco **(B)**], 19 Hex-fucoxanthin [Hex-Fuco **(C)**], Zeaxanthin [Zea **(D)**], Chlorophyll *b* [Chl*b* **(E)**], and Total Chlorophyll *a* [Chl*a* **(F)**] are shown.

Using the distribution of the relative biomarker pigment concentrations (normalized against Chl*a*), dominating phytoplankton groups can be identified (Jeffrey and Wright, 2006). Maximum Chl*a* associated with larger phytoplankton correlated with total Chl*a* distribution (Figures 6 and 7). Large phytoplankton, present at the depth of Chl*a* maximum were mainly diatoms in the W-SAZ and PFZ (as indicated by a clear dominance of fucoxanthin) and were mainly haptophytes in the E-SAZ (dominance of 19-hexanoyloxyfucoxanthin, Figure 7). In the PFZ and E-SAZ, the Chl*a* associated with small phytoplankton correlated with total Chl*a* maximum (Figures 6 and 8). In the W-SAZ, most of the S-phyto were present at 50 m, below the total Chl*a* maximum (Figure 8). Haptophytes and diatoms were mainly present in the subantarctic water west of Tasmania (Stn 2). In the W-SAZ (Stn 4), haptophytes, and green algae (Chl*b*) dominated in surface water; some cyanobacteria (zeaxanthin and Chl*b*) and diatoms were also present at 50 m. At the depth of total Chl*a* maximum, small diatoms, and haptophytes dominated in the PFZ, whereas cyanobacteria and diatoms dominated in the E-SAZ. Peridinin was only significantly present in the E-SAZ (data not shown), representing 24 and 8% of large and small phytoplankton Chl*a*, respectively. Prasinoxanthin was only present at marginal levels (1% of Chl*a*) at Stns 12 and 17 (data not shown).

**Figure 9:**
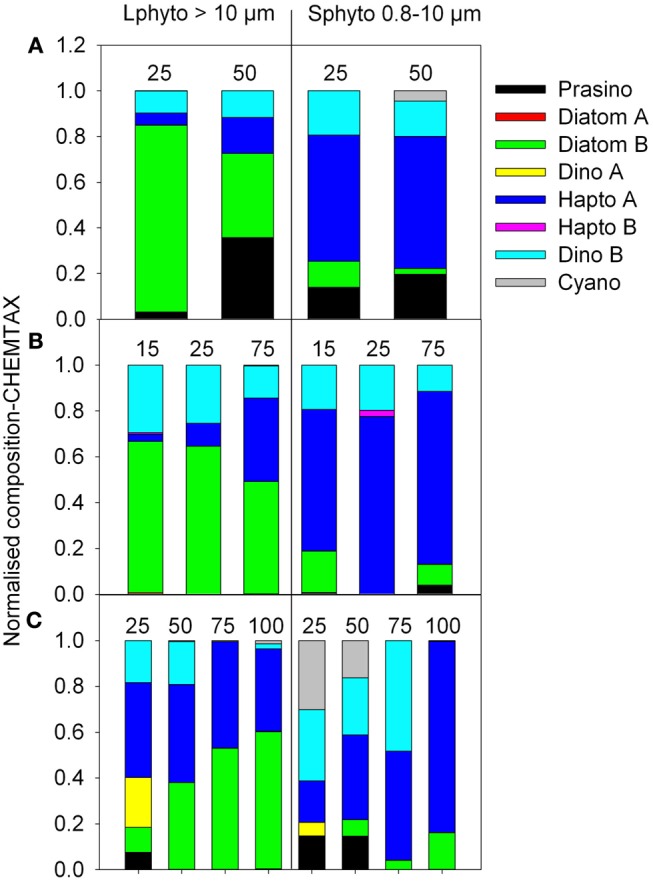
**Estimation of the phytoplankton community using CHEMTAX per de Salas et al. (2011)**. The major groups composing the small (S, 0.8–10 μm) and large (L, >10 μm) phytoplankton communities are represented in percent (stacked bars) at different depths for stations in the E-SAZ [Stn 4 **(A)]**, PFZ [Stn 6 **(B)**], and W-SAZ [Stn 17 **(C)**].

The use of CHEMTAX revealed that, except for prasinophytes, representing up to 35% of the large and small phytoplankton in the W-SAZ, the composition of the phytoplankton community calculated by CHEMTAX was similar in the W-SAZ and the PFZ (Figures 9A,B). In these two zones, large phytoplankton were dominated by diatoms, whereas small phytoplankton were dominated by haptophytes. Dinoflagellates were present in both phytoplankton size fractions, representing 10–30% of the phytoplankton community. In the E-SAZ, the composition of the phytoplankton community inferred by CHEMTAX was more complex (Figure 9C). In the phytoplankton bloom (top 40 m, Figure 6), diatoms are only marginal (10%, Figure 9C), likely due to Si depletion. Large phytoplankton are mainly composed of dinoflagellates and haptophytes (40% each), whereas small phytoplankton mainly contained dinoflagellates (37%), and cyanobacteria (30%), with some haptophytes (18%) and prasinophytes (14%).

### Statistical correlations of nutrients with physical and biological parameters

**Figure 10:**
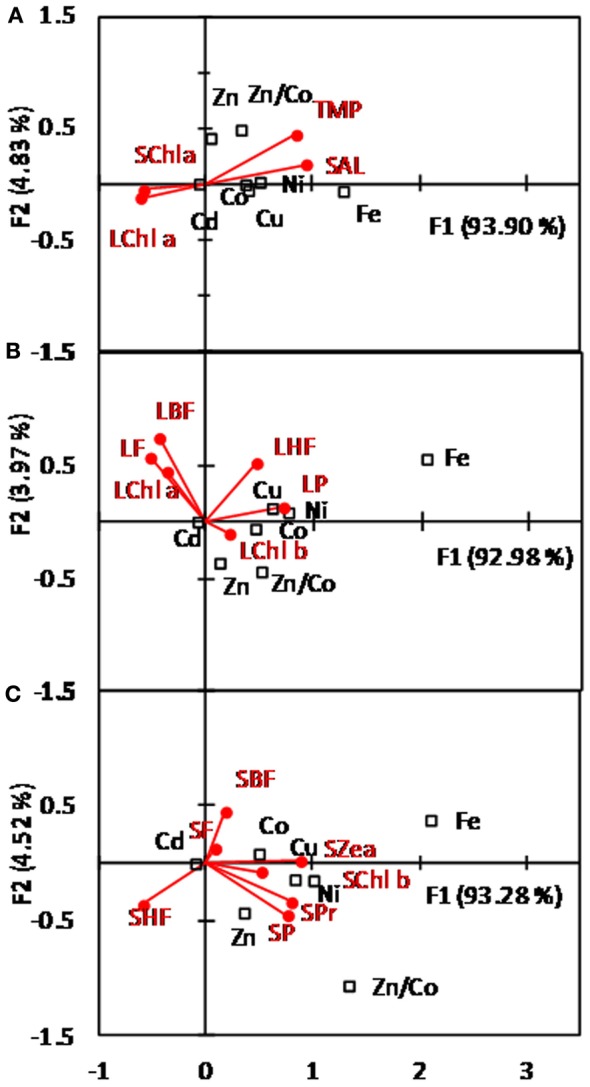
**Symmetric CCA map between micronutrients defined as objects (square) and physical and biological variables (red dots)**. **(A)** considers micronutrients with the physical variables temperature (TMP) or salinity (SAL) and the variable chlorophyll (Chl *a*) from the large (L, >10 μM) or small (S, 0.8–10 μM) phytoplankton community. Relations between micronutrients with the biomarker pigments associated with the large (L) and small (S) phytoplankton community are represented in **(B)** and **(C)**, respectively. Micronutrients are cobalt (Co); nickel (Ni); copper (Cu); zinc (Zn); cadmium (Cd); iron (Fe). Biomarker pigments are Chlorophyll (C); Peridin (P); 19-But-Fucoxanthin (BF); Fucoxanthin (F); 19-Hex-fucoxanthin (HF); Prasinoxanthin (Pr); zeaxanthin (Zea). Data for Fe are from Lannuzel et al. (2011).

Canonical correspondence analysis representation of micronutrients according to temperature, salinity, small, and large phytoplankton Chl*a* (Figure 10A, 0–100 m), demonstrated four micronutrient “clusters”, with Fe being closer to physical parameters and Cd closer to Chl*a*. The other micronutrients were intermediately located with Cu, Co, and Ni closer to physical parameters and Zn closer to Chl*a*. These data were also supported by the Pearson’s correlation tests (Table A1 in Appendix). Indeed, Fe was the only micronutrient to significantly positively correlate with temperature and salinity but not significantly with NO_x_ and PO_4_. No significant correlation between micronutrients and silicic acid were obtained. Co, Cu, Ni, Cd, and Zn were significantly positively inter-correlated and all negatively correlated with Fe. Fe was also significantly negatively correlated to ratio dZn/dCo (Table A1 in Appendix).

**Figure 11:**
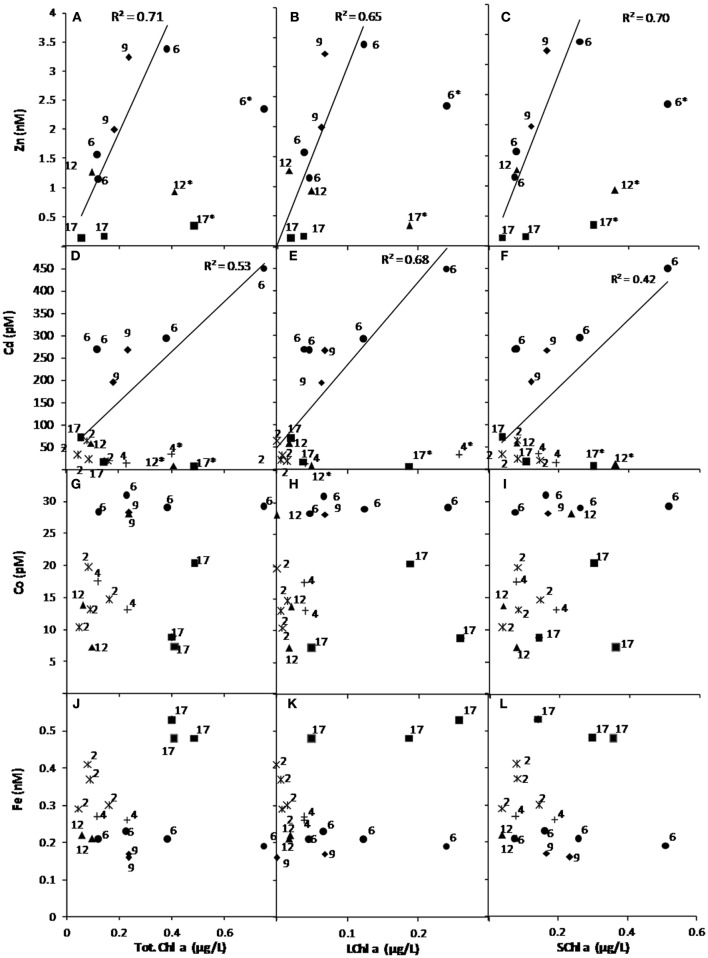
**Relation between dissolved micronutrients and chlorophyll *a* associated with the total [Chl*a*, (A), (D), (G), (J)], large [LChl*a*, >10 μm, (B), (E), (H), (K)], and small [SChl*a*, 0.8–10 μm, (C), (F), (I), (L)] phytoplankton communities**. Relationships are shown for Zn **(A–C)**, Cd **(D–F)**, Co **(G–I)** and Fe **(J–L)** and coefficient of correlation are given when significant relationships were found (Zn and Cd only). Each station has been represented using a different symbol and the station labeled with “*” correspond to depth with a maximum of Chl*a* and were removed to obtain a significant correlation for Cd and Zn. Fe data are from Lannuzel et al. (2011).

No statistically significant Pearson’s coefficient of correlation was observed between micronutrients and Chl*a* throughout the study region (total, large, and small phytoplankton, Table A1 in Appendix). However, the degree of significance of positive correlation followed the order Cd > Zn > Ni, Cu, Co, with Ni and Cu more related to small phytoplankton Chl*a*. Fe was only weakly correlated (−0.1) to Chl*a*. These correlations are represented for Cd, Zn, Co, and Fe in Figure 11. Data from the depths of Chl*a* maximum (labeled as “*” in Figures 11A–F) significantly deviated from dCd/Chl*a* and dZn/Chl*a* relations. Exclusion of these points, resulted in a significant correlation between dissolved Cd and Zn with total, small, and large Chl*a* concentrations. No correlation was observed between Co, Fe, and Chl*a* (Figures 11G–L).

Canonical correspondence analysis representations of micronutrients with large (L- Figure 10B) and small (S- Figure 10C) phytoplankton biomarker pigments confirmed that Fe was poorly related to phytoplankton. Fe was closest to L-peridinin and S-zeazanthin, the only two pigments for which a significant positive correlation was obtained (Table A1 in Appendix), suggesting that Fe could affect the dynamics of dinoflagellates and cyanobacteria in the E-SAZ. Cd was the micronutrient that was the most closely related to all biomarker pigments (Figures 10B,C), whereas Cu and Ni formed a cluster closely related to L-peridinin, L-hex-fucoxanthin, and S-zeazanthin, S-prasioxanthin, S-Chl*b*, and S-peridinin. Co and Zn were differentiated by Co being closer to L-peridinin and L-Chl*b*, S-fucoxanthin, S-but-fucoxanthin, S-Chl*b* and S-zeazanthin, and Zn being closer to L-Chl*b* and S-peridinin, S-prasinoxanthin and S-Chl*b*. The ratio dZn/dCo was more associated with biomarker pigments from large rather than small phytoplankton. Co, Ni, Cu, and Cd were statistically positively correlated with L-but-fucoxanthin, L-fucoxanthin, and negatively correlated to S-Chl*b*. Co was also significantly negatively correlated to S-zeaxanthin. Zn is only significantly positively correlated with Shex-fucoxanthin and dZn/dCo to L-hex-fucoxanthin (Table A1 in Appendix). Pearson’s correlation tests also showed significant positive correlations between NO_x_ and PO_4_ with L-but-fucoxanthin and L-fucoxanthin, between Si and S-fucoxanthin, and between nitrite (NO_2)_ and S-hex-fucoxanthin (Table A1 in Appendix).

## Discussion

Previous studies from the SAZ-Sense project have demonstrated that the E-SAZ, despite greater Chl*a* was less productive than the W-SAZ at the time of observation (Cavagna et al., 2011; Westwood et al., 2011; Table A1 in Appendix). Biomarker pigments and CHEMTAX analysis showed a contrasted phytoplankton community in the study region. Distribution of phytoplankton size classes and dominant groups accorded with microscopic observations, pigment size class, and CHEMTAX calculations done during the SAZ-Sense voyage (de Salas et al., 2011; Pearce et al., 2011). There is, therefore, a clear difference between these two SAZ regions.

Our distribution of micronutrients agreed with previous studies in the region (Ellwood, 2008; Lai et al., 2008). Similar hydrology for these studies, despite the difference in season and location (winter and eastwards of the SAZ-Sense study for Ellwood, 2008), allow a comparison of the distribution of micronutrients. Similar Cd, Cu, and Ni distribution in the SAZ, closely related to PO_4_ (see Figure 5), suggested that this distribution of micronutrients, is the result of long term processes (seasonal and longer – see also Butler et al., in revision). Co, too, was closely related to PO_4_ in the top 150–200 m and with a distribution that tracks with timescales of seasonal or longer. Deeper in the SAZ water column, Co is known to display scavenged characteristics, and is strongly influenced by concentrating processes in the Antarctic Zone (seen to some degree here at Stns 6, 9, and 10) and intermediate water formation (Butler et al., submitted). By comparison, the dissolved iron distribution resulted from short term processes, involving a rapid recycling, episodic dust input, and intrusion of oligotrophic subtropical water into the SAZ region, generating a patchy distribution of dissolved iron (Bowie et al., 2009, 2011a,b; Lannuzel et al., 2011; Mongin et al., 2011). Butler et al. (in revision) have also proposed that distribution of Zn in SAZ-E waters was modulated by short term processes at the time of the SAZ-Sense voyage, although it was quite unlike that of Fe, which is supported by their lack of correlation reported here. The patchiness of Fe and Zn at SAZ-E results from region being dominated by the East Australian Current eddy field, the different supply mechanisms for the two pivotal metals, and the ensuing sub-mesoscale irregularities in phytoplankton productivity

Determination of the control by the full suite of micronutrients (Fe, Zn, and other transition metals) on phytoplankton biomass and community structure is, thus, first step to elucidate how primary productivity and the balance between carbon export and recycling are controlled. However, the identification of the nutritive status (e.g., limited, co-limited, replete) of *in situ* phytoplankton communities is a major challenge. Statistical analyses of the relationship between micronutrients and phytoplankton, revealed complex heterogeneous relationships in which all micronutrients (Fe, Zn, Cd, Co, Cu, and Ni) are important for specific phytoplankton groups. All the micronutrients studied were depleted in surface waters and presented good correlations with dissolved PO_4_ (except Fe), demonstrating their nutritive role in phytoplankton growth. The role of micro-nutrients in shaping the phytoplankton community can be further explored by comparing their disappearance ratios in reference to PO_4_ with the dissolved metal spot ratios, with previously reported phytoplankton cellular quotas and biological requirements (e.g., Sunda and Huntsman, 1995a,b; Ho et al., 2003; Finkel et al., 2007; Saito et al., 2010; Croot et al., 2011).

In surface water, a greater disappearance ratio is often associated with a greater relative biological uptake but could also be related to differential recycling and remineralization. Several studies have reported a “kink” in the disappearance ratio for Cd which, despite a poor understanding of the mechanisms at play, can be related to a decreased phytoplankton growth rate or “biodilution effect” (Sunda and Huntsman, 2000; Cullen et al., 2003). It is, thus, not surprising that, in the CCA, Cd was the closest to total Chl*a* and to most of the biomarker pigments. Cadmium was the most depleted micronutrient in surface waters (from comparison with concentrations at 150–200 m depth – see Bowie et al., 2011b) and it also showed significant correlation with several biomarker pigments. The disappearance ratio for Cd observed here was slightly lower than for the Ross Sea and the subarctic Pacific (Table 3).

In this study, kinks in disappearance ratios were observed for NO_x_, Zn, and Cd. The kinks observed for NO_x_ and Cd correspond to the surface water (25–50 m) of the W- and E-SAZ (Stns 2,4, and 17) but the kink observed for Zn extends deeper at these stations and further south to Stns 5 and 12, suggesting a complex control on phytoplankton growth rate in the region. This is emphasized by the fact that inter-station disappearance ratio is variable and follows a general pattern, where disappearance ratios are increasing southwards with a loss of correlation in the PFZ. Such loss of correlation could be due to nutrients supply from Upper Circumpolar Deep Water sufficient to swamp the imprint of biological uptake. On the other hand, the disappearance ratio for NO_x_ is lower in the PFZ. In the Southern Ocean, such a poleward increase in disappearance ratio was recently reported for Zn (Croot et al., 2011), whereas a lower dNO_x_/PO_4_ was reported in the PFZ and southwards (Levitus et al., 1993; de Baar et al., 1997). These observations were attributed to variation in phytoplankton growth rate and specific phytoplankton uptake ratio (de Baar et al., 1997; Arrigo et al., 1999; Croot et al., 2011). Parameters usually discussed as a control in phytoplankton growth rate with impact on disappearance ratios in the region are (i) iron limitation (e.g., Martin et al., 1990; de Baar et al., 1997; Cullen et al., 2003; Twining et al., 2004a,b; Croot et al., 2011), (ii) combined effect of micronutrients (Sunda and Huntsman, 2000; Twining et al., 2004b; Cullen and Sherrell, 2005), and (iii) community structure (e.g., Sunda and Huntsman, 1995b, 2000; de Baar et al., 1997; Arrigo et al., 1999; Ho et al., 2003; Quigg et al., 2003; Twining et al., 2004a,b; Finkel et al., 2010). It is to be noted that the dependency of disappearance ratios on growth rate and iron addition is not an invariant attribute; it can differ between phytoplankton groups and micronutrients (e.g., Twining et al., 2004b; Finkel et al., 2007). Finally, the effects of light and temperature have also to be considered (Finkel et al., 2007, 2010; Croot et al., 2011).

Comparison of the distribution of the Chl*a* (Figure 6) with the depths of the euphotic zone and the mixed layer (Table A1 in Appendix) allows discussion of the impact of light limitation. In both the W-SAZ and PFZ, most of the Chl*a* is found at the bottom of the euphotic layer, just below the MLD. This clearly illustrates a trade-off between light and nutrient limitation at these sites. In the E-SAZ, light might constrain the bloom in surface waters (Mongin et al., 2011; Westwood et al., 2011; Table A1 in Appendix), but phytoplankton found in the top 16 m are not light limited. Temperature is known to exert a control in the growth of cyanobacteria (Li, 1998) and can, thus, curtail their extension southwards. However, temperature cannot account for the absence of cyanobacteria in the W-SAZ. In addition, as previously noted, it is likely that phytoplankton present at these high latitudes are well adapted to low temperatures (e.g., Croot et al., 2002). Therefore, it is not expected that temperature acts as a fundamental control of phytoplankton growth here.

During SAZ-Sense, Fe, and Si limitation were postulated in the W-SAZ and the PFZ using microscopic observations (Pearce et al., 2011), diatom silification rate (Fripiat et al., 2011), Si*, and Fe* concentrations (Lannuzel et al., 2011), incubation and Fv/Fm (Schoemann et al., unpublished). Interestingly, except for prasinophytes, the composition of the phytoplankton community was similar at these two locations. The lower disappearance ratio for NO_x_ in the PFZ could be related to diatoms, such as *Fragilariopsis kerguelensis*, present at Stn 9 (de Salas et al., 2011), and iron limitation (de Baar et al., 1997). By comparison, in the E-SAZ, where a less productive but more complex and diverse phytoplankton community prevailed (this study, de Salas et al., 2011), no iron limitation was observed. In the surface waters of the E-SAZ, the decreased dZn/dCo and dZn/dCd likely reflected biological uptake, whereas the increased dFe/dCo and dFe/dZn likely reflected the iron aerosol input (Bowie et al., 2009; Mongin et al., 2011). The dissolved Fe/PO_4_ ratio also indicated iron enrichment in the surface waters of the W-SAZ and E-SAZ. Based on the iron biological requirement for phytoplankton growth (i.e., half saturation constant), the dissolved iron concentrations measured in surface water were indeed enough to sustain the growth of most diatoms (Coale et al., 2003; Sarthou et al., 2005), and the haptophyte genus, *Phaeocystis* (Coale et al., 2003; Sedwick et al., 2007). However, diatoms were a marginal phytoplankton group, and nanoflagellates and dinoflagellates were the most dominant groups in the E-SAZ at the time of study (this study; de Salas et al., 2011; Pearce et al., 2011). Dinoflagellates were previously reported as dominating the phytoplankton community in the SAZ region south of Australia during austral spring and summer (Kopczynska et al., 2001, 2007). Studies of elemental ratios in phytoplankton have demonstrated that optimal iron content, follows the order cyanobacteria > haptophytes ≥ dinoflagellates ≥  diatoms (Ho et al., 2003; Twining et al., 2004b; Finkel et al., 2010) and that iron enrichment favors the growth of diatoms, but also autotrophic and heterotrophic flagellates (e.g., Twining et al., 2004a). In this case, additional iron sources in the E-SAZ might have relieved iron limitation and favored the growth of flagellates and cyanobacteria. Indeed, dissolved iron concentrations were significantly related to L-Peridin (dinoflagellates – type 1) and S-zeaxanthin (cyanobacteria), both biomarkers present only in the E-SAZ. In this case, phytoplankton in the E-SAZ were not iron-limited at the time of sampling; however, iron had an important effect of the phytoplankton community structure in this region.

The extended Redfield ratio based on micronutrient cellular quota from phytoplankton cultures of P_1_Fe_7.5_Zn_0.80_Cu_0.38_Co_0.19_Cd_0.21_ (Ho et al., 2003), suggests that the biological requirement for growth is usually smaller for other micronutrients than for iron. However, a significantly different extended Redfield ratio of P_1_Zn_5.4_Fe_1.8_Ni_0.61_ was calculated from a field study during the Southern Ocean Iron Experiment (SOFeX, Twining et al., 2004b), suggesting that Zn biological requirement exceeds the requirement for iron. These differences could be related to different water chemistry, light and phytoplankton species as all of these parameters affect cellular quotas (Finkel et al., 2010). In the study region, the concentrations of micronutrients are lower than iron, especially in the SAZ regions, suggesting that they might play a role in controlling phytoplankton growth or community structure.

Cd and Co nutrition in phytoplankton is coupled to Zn nutrition, because Zn can interchange with Cd and Co to support key enzymes, such as the carbonic anhydrase in some (Price and Morel, 1990; Morel et al., 1994; Sunda and Huntsman, 1995a, 2000; Saito and Goepfert, 2008), but not all phytoplankton species (Timmermans et al., 2001). Laboratory studies suggested that Cd, Co, and Zn use the same biological transport system, which is regulated by cellular Zn concentrations for diatoms and coccolithophores (Sunda and Huntsman, 1995a, 2000). Cobalt is associated with vitamin B_12_ which is essential to, but not synthesized by eukaryotic phytoplankton (Saito et al., 2002; Saito and Goepfert, 2008).

**Figure A2:**
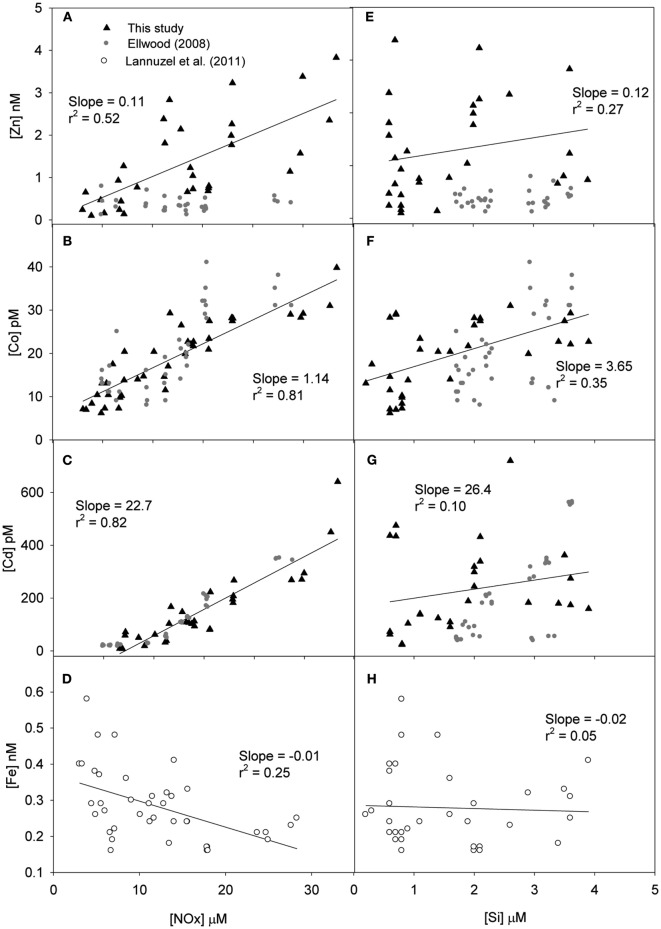
**Disappearance ratio of dissolved micronutrients (Zn, Co, Cd, Fe) with dissolved NO_x_ (NO_2_ + NO_3_, panels, A, B, C, D, respectively) and silicic acid (Si, panels E, F, G, H, respectively) for the top 100 m for the Subantarctic and Polar Frontal Zone (Australian sector)**. Depths and stations are not differentiated. The slope and the coefficient of correlation for linear regression are shown. Data from a previous study in the region is also shown (Ellwood, 2008). It is to be noted that Ellwood (2008) did not cross the North Polar Front into Antarctic Zone, which could explain some discrepancy. Data for Fe are from Lannuzel et al. (2011).

The kink observed for dZn/PO_4_, could be related to the induction of high affinity transporters which are efficient in maintaining a nearly constant cellular Zn concentration despite decreasing free Zn concentrations (Sunda and Huntsman, 1995a). Zn was the only micronutrient for which significant differences in the relation with other macronutrients were observed (Figure 5; Table 3). In Ellwood (2008), dissolved Zn and PO_4_ concentrations were not significantly related in surface waters (*r*^2^ < 0.001, 0–100 m, see Figure A1 in Appendix) but dissolved Zn and Si were strongly related (*r*^2^ = 91). Our data suggest a greater role for Zn in phytoplankton nutrition. The lack of dZn/Si relationship observed here (Figure A2 in Appendix) could be related to diatoms not being the dominant phytoplankton group in the SAZ at the time set of our study (de Salas et al., 2011), likely related to Si depletion and limitation (Fripiat et al., 2011).

Here, the ratios dZn/PO_4_:dCd/PO_4_ of 5 and dZn/PO_4_: dCo/PO_4_ of 96 (Table 3) showed indeed that Zn had an important nutritive role in the study region. The Zn/PO_4_ measured here is much higher than data previously reported (Table 3, except for Croot et al., 2011). The dZn/PO_4_ ratio observed here (>0.5 μM PO_4_) was close to the cellular ratio for which growth inhibition is observed in *Emiliania huxleyi* (Table 3, in absence of Co and Cd, Sunda and Huntsman, 1995a). For a similar Zn^2+^/Cd^2+^ than the dZn/dCd ratio observed here, the cellular Zn:P ratio in *E. huxleyi* (1494 μmol mol^−1^; Sunda and Huntsman, 2000) was close to the dZn/PO_4_ ratio observed. This suggests that Zn was present at concentrations which could limit or co-limit the growth of coccolithophores, even considering Zn-Cd biological substitution. However, in the E-SAZ, the ratios between Zn and Co dissolved concentrations were smaller than 96, suggesting that Co could be important to complement Zn nutrition. The disappearance ratio for Co was smaller than the cellular Co:P ratios under which growth inhibition was observed for diatoms and coccolithophores (Table 3), suggesting that phytoplankton in the E-SAZ could be limited or co-limited by Co.

Based on the Pearson’s correlation coefficient, dissolved Zn concentrations were related to L-Chl*b*, S-Chl*b*, S-peridinin, and S-prasinoxanthin, biomarker pigments mostly present in the E-SAZ and at depth in the W-SAZ (50 m), suggesting that Zn nutrition is important in these regions. This observation is reinforced by the fact that higher dissolved ecological stoichiometry was observed at Stns 9 and 10 and that data from the depth of the Chl maximum had a Zn and Cd concentration much lower than those predicted from the dZn/Chl*a* and dCd/Chl*a*. Co was significantly related to L-peridinin, L-Chl*b*, S-Chl*b*, S-fucoxanthin, S-but-fucoxanthin, and S-zeaxanthin. Because these biomarker pigments are mainly present in the W-SAZ (Stn 4, 50 m) and in the E-SAZ (Stns 10–17), it suggests that Co could shape the structure of the phytoplankton community in the SAZ region. The effect of Co on S-Chl*b* and S-zeaxanthin is not surprising given that studies have demonstrated a strict Co requirement in cyanobacteria (Sunda and Huntsman, 1995a; Saito et al., 2002). Oceanic prasinophytes and dinoflagellates, the dominating phytoplankton in the SAZ region, have a greater requirement in Zn, Co, and Cd than diatoms (Ho et al., 2003). Laboratory work demonstrated that the haptophyte, *Phaeocystis*, and diatoms prefer Zn to satisfy their growth requirement, while the haptophyte, *E. huxleyi* prefers Co (Sunda and Huntsman, 2000; Saito and Goepfert, 2008). An elevated dZn/dCo ratio, could favor the growth of diatoms and *Phaeocystis* and be a disadvantage for the growth of *E. huxleyi* (Sunda and Huntsman, 1995a; Saito and Goepfert, 2008). In this study, dZn/dCo was more strongly related to hex-fucoxanthin a biomarker for haptophytes and dinoflagellates (type 2), but not significantly related to fucoxanthin. This might be because fucoxanthin is not only present in diatoms, but also in haptophytes, chrysophytes, and some dinoflagellates (Jeffrey and Wright, 2006). It should be noted that, in the field, the preference for Zn or Co for natural phytoplankton communities remains largely unknown (e.g., Croot et al., 2002).

Iron is also known to affect the biological uptake of other micronutrients (e.g., Cullen et al., 2003; Ho et al., 2003; Twining et al., 2004b; Cullen and Sherrell, 2005). It, therefore, appears that complex interconnections exist between these metals and phytoplankton. In fact, a significant negative correlation for dFe with dZn/dCo and a high coefficient of correlation (although not statistically significant, 0.4) with hex-fucoxanthin, suggest an interaction between Fe, Zn, and Co in the control of the phytoplankton community in the SAZ region.

This study also focuses on other micronutrients for which data are yet limited. Both Cu and Ni had the same pattern of correlations with biomarker pigments; however, little is known of a coupled effect for Cu and Ni on phytoplankton growth. Both these micronutrients were significantly correlated to L-peridinin, S-peridinin, L-hex-fucoxanthin, S-zeaxanthin, S-prasinoxanthin, S-Chl*b*, demonstrating an effect on most major phytoplankton groups, except diatoms. Cu is involved in nitrogen cycling, electron transfer associated with photosynthesis and Fe uptake (Morel et al., 2003; Maldonado et al., 2006), and Ni is involved in urea assimilation and protection against reactive oxygen species (Morel et al., 2003; Dupont et al., 2010). The disappearance ratios for Ni and Cu are close to previously reported values (Table 3). Comparison of the dCu/PO_4_ with cellular Cu:P ratio for *Thalassiosira oceanica* and *E. huxleyi* (Table 3), as well as other oceanic species including dinoflagellates (Cu:P 60–110 μmol:mol, Ho et al., 2003), suggests that Cu was present at an optimal level for the growth of these eukaryotic phytoplankton. For dCu, a relation with PO_4_ (*r*^2^ > 0.75) was only observed for stations within the W-SAZ and E-SAZ, suggesting a nutrition role in these regions. For Ni, the disappearance ratio measured here (Table 3) was also greater than cellular Ni:P ratios for diatoms and flagellates (160–1150 μmol:mol, Twining et al., 2004b), suggesting optimal conditions for growth in the study region.

As a cautionary note, the disappearance ratios used here can be influenced by scavenging processes, surface input, organic complexation, recycling, and remineralization, biological uptake, and drawdown (e.g., Saito et al., 2010; Croot et al., 2011). Recent studies highlighted important differences in Zn organic speciation between the SAZ and the PFZ, with greater inorganic Zn concentration found in the PFZ (e.g., Ellwood, 2004; Baars and Croot, 2011). For micronutrients that are influenced by short time processes (such as Fe), it is expected that this relation (metal to PO_4_) will be significantly blurred, and less meaningful. In addition, snapshot studies will invariably fail to represent the dynamic situation between micronutrients and phytoplankton. For example, grazing, recycling, supply, and phytoplankton succession are not considered. During SAZ-Sense, regenerated production exceeded new production (Cavagna et al., 2011) and grazing dilution experiments demonstrated the complexity of the processes at play with on average 36–37% herbivory, 39–42% bacterivory, and 21–24% cyanobacterivory (Stns 4 and 17; Pearce et al., 2011). Grazing is paramount for nutrient recycling, which was very efficient for Fe in the E-SAZ (Bowie et al., 2009). Nonetheless, our study provides much needed information on the biological relevance of micronutrients, other than iron during the austral summer of 2007. It indicated that Zn, Co, and Cd inter-replacement was influential in the E-SAZ region and that interaction between these micronutrients and Fe needs to be considered. Our results also demonstrated that different phytoplankton size classes and functional groups are related differently to micronutrients, possibly attributed to variable biological requirements for growth. Generally, larger phytoplankton have greater micronutrient requirement for growth (Sarthou et al., 2005; Hassler and Schoemann, 2009; Finkel et al., 2010). Here, L-phyto were not more strongly related to micronutrients than S-phyto, illustrating the complexity at play in the field.

If the E-SAZ, represents the future of the SAZ region, further investigations on the relations among Fe, Cd, Zn, and Co and their ability to shape the structure of the phytoplankton community is required. In addition, effort should be made to increase our knowledge of the micronutrient requirement for dinoflagellates, a dominant group in the E-SAZ. To integrate the key processes at play, ship-board experiments would be required to study the effect of micronutrients on the natural phytoplankton community and measure their requirement for growth (e.g., Coale et al., 2003). Because macro- and micronutrients support biological processes that are interrelated or have synergistic or antagonistic relations, an experimental approach at sea should investigate these complex interactions, rather than focusing on a single element.

## Conflict of Interest Statement

The authors declare that the research was conducted in the absence of any commercial or financial relationships that could be construed as a potential conflict of interest.

## Footnotes

^1^www.geotraces.org/libraries/documents/Intercalibration/Cookbook.pdf

## Appendix

**Table A1:** **Summary of important properties from the W-SAZ (Stn 4), PFZ (Stn 6), and E-SAZ (Stn 9) during the SAZ-Sense study**.

Properties	W-SAZ	PFZ	E-SAZ
Euphotic layer depth^1^, m	61 ± 5	76 ± 14	47 ± 13
Mixed layer depth^2^, m	41 ± 18	53 ± 6	79 ± 2 (16 ± 2)
Integrated Chl *a*^3^	46.0 ± 11.5	58.8 ± 2.9	62.4 ± 20.0
Gross primary production^4^	93 ± 49	37 ± 7	60 ± 29
Grazing rate^5^	82 ± 38	47 ± 10	67 ± 12
Remineralization rate^6^	2.1 ± 0.4	5.0 ± 1.6	3.7 ± 0.4
Nitrate seasonal depletion^7^	8.9 ± 1.8	8.1 ± 0.4	11.0 ± 1.7
N:P seasonal depletion ratio^8^	16 ± 6	12 ± 1	17 ± 3

*^1^From Westwood et al. (2011), ^2^Calculated after Rintoul and Trull (2001), a distinct shallow mixed layer depth is shown in bracket for the E-SAZ, see Mongin et al. (2011), ^3^0–150 m (mg m^−2^), from Westwood et al. (2011), ^4^mmol Cm^–2^ d^−1^, from Cavagna et al. (2011), ^5^% Total Primary Production removed, from Pearce et al. (2011), ^6^Mesopelagic C remineralization, 100–600 m (mmol Cm^−2^ d^−1^) from Jacquet et al. (2011), ^7^Mixed layer N depletion uses means from 150 to 200 m depth to represent winter surface values (mmol L^−1^) from Bowie et al. (2011b), ^8^Mixed layer N:P ratio uses means from 150 to 200 m depth to represent winter surface values from Bowie et al. (2011b)*.

**Table A2:** **Metal disappearance ratio relative to dissolved phosphate for each station**.

Stn #	Latitude (°S)	Longitude (°E)	Co	Zn	Cd	Ni	Cu
2	44.89	143.05	20.4	NA	147	1.52	ND
			(0.92)		(0.52)	(1.00)	*0.28*
			*11.0*		*433*	*1.67*	*(0.85)*
			*(0.54)*		*(0.99)*	*(0.91)*	
4	46.32	140.61	13.6	NA	159	1.12	ND
			(0.84)		(0.74)	(0.60)	*0.63*
			*12.5*		*486*	*1.84*	*(0.95)*
			*(0.58*)		*(0.96)*	*(0.95)*	
5	48.99	143	106	NA	871	5.50	0.27
			(0.98)		(1.00)	(0.96)	(0.76)
			*7.02*		*379*	*1.70*	*0.31*
			*(0.53)*		*(0.99)*	*(0.98)*	*(0.87)*
6	54.00	145.88	ND	4.73	489	0.73	0.72
			*ND*	(0.60)	(0.92)	(0.73)	(0.71)
				*7.38*	*463*	*1.58*	*1.31*
				*(0.86)*	*(0.90)*	*(0.95)*	*(0.76)*
9	50.87	148.65	ND	ND	ND	ND	−2.55
			*79.4*	*ND*	*258*	*4.73*	(0.45)
			*(0.72)*		*(0.67)*	*(0.40)*	*ND*
10	50.00	149.42	180	8.36	ND	ND	ND
			(0.42)	(0.50)	*931*	*ND*	*ND*
			*ND*	*7.64*	*(0.84)*		
				*(0.41)*			
12	48.06	151.20	46.8	5.77	456	1.70	0.62
			(0.90)	(0.48)	(0.97)	(0.57)	(0.78)
			*59.2*	*ND*	*451*	*1.32*	*0.29*
			*(0.70*)		*(0.76)*	*(0.64)*	*(0.34)*
17b	45.55	153.18	20.6	0.99	84.9	1.46	0.35
			(0.80)	(0.46)	(0.86)	(0.91)	(0.98)
			*5.00*	*1.29*	*346*	*1.92*	*0.35*
			*(0.32)*	*(0.59)*	*(0.98)*	*(1.00)*	*(0.91)*
17j	45.55	153.18	24 (0.73)	0.73	142	1.08	0.24
			*6.17*	(0.54)	(0.86)	(0.72)	(0.72)
			*(0.62)*	*0.61*	*371*	*1.78*	*0.36*
				*(0.83)*	*(0.94)*	*(0.98)*	*(0.92)*

*Disappearance ratios are presented with their coefficient of correlation (*r*^2^, in bracket) for depths between 0 and 100 m (roman font, *n* = 4) and 125–1000 m (italic font, *n* = 7). Disappearance ratio is given in pM/μM for Co, Cd, and Ni, and in nM/μM for Cu and Zn. NA = not applicable due to lack of data, ND = not detected as *r*^2^ < 0.3*.

**Table A3:** **Pearson correlation table showing the coefficient of correlation between two parameters considering all stations and all depths (0–125 m)**.

Variables	T															
S	**0.92**	S														
SI	0.23	0.27	SI													
NOx	**−0.76**	**−0.75**	0.19	NOx												
Ph	**−0.79**	**−0.76**	**−**0.28	**0.98**	Ph											
A	**−**0.29	**−**0.17	**−**0.05	0.00	0.12	A										
N	**−**0.44	**−**0.43	**−**0.32	0.52	0.54	0.45	N									
Co	**−0.90**	**−0.90**	**−**0.09	**0.85**	**0.85**	0.15	**0.81**	Co								
Ni	**−0.97**	**−0.95**	0.07	**0.86**	**0.86**	0.15	**0.75**	**0.90**	Ni							
Cu	**−0.93**	**−0.84**	0.15	**0.77**	**0.78**	0.25	**0.72**	**0.77**	**0.91**	Cu						
Zn	**−**0.56	**−0.68**	0.12	0.73	**0.84**	0.59	**0.81**	**0.72**	**0.63**	0.51	Zn					
Cd	**−0.96**	**−0.92**	0.08	**0.78**	**0.80**	0.13	0.69	**0.90**	**0.93**	**0.82**	**0.75**	Cd				
Fe	**0.56**	**0.73**	0.28	**−**0.51	**−**0.50	**−**0.13	**−0.71**	**−0.55**	**−0.54**	**−**0.40	**−0.77**	**−0.55**	Fe			
Zn/Co	**−**0.31	**−**0.44	**−**0.08	0.04	0.16	0.64	0.49	0.13	0.14	0.08	**0.71**	0.23	**−0.74**	Zn/Co		
Tot Chl	**−0.66**	**−**0.50	**−**0.21	**−**0.14	**−**0.05	**0.60**	**−**0.15	0.14	0.26	0.17	0.30	0.40	**−**0.14	0.35	Tot Chl a	
LChl	**−0.79**	**−0.66**	**−**0.26	**−**0.11	**−**0.05	0.27	**−**0.36	0.15	0.19	0.02	0.30	0.35	**−**0.08	0.12	**0.84**	LChl a
SChl a	**−0.56**	**−**0.39	**−**0.07	**−**0.12	**−**0.03	**0.66**	0.10	0.11	0.27	0.25	0.29	0.38	**−**0.17	0.45	**0.94**	**0.60**

*The significance of the correlation is based on the *p* value (*p* > 0.05) obtained for a 95% confidence interval (data not shown). Values in red are significantly negatively correlated in and values in yellow are significantly positively correlated. The correlation between physical properties (temperature, T, and salinity, S), macronutrients, micronutrients, and the chlorophyll a (Chl a) associated with the total, large (L, >10 μm) and small (S, 0.8–10 μm) phytoplankton communities were analyzed. Macronutrients are: silicic acid (SI); NO_2_ + NO_3_ (NOx); phosphate (Ph); ammonia (A); nitrite (N). Micronutrients are: cobalt (Co); nickel (Ni); copper (Cu); zinc (Zn); cadmium (Cd); iron (Fe). Fe data are from Lannuzel et al. (2011)*.

**Table A4:** **Pearson correlation table showing the coefficient of correlation between two parameters considering all stations and al depths (0–125 m)**.

Variables	SI															
NOx	**0.19**	NOx														
Ph	0.28	**0.98**	Ph													
A	**−**0.05	0.00	0.12	A												
N	**−0.32**	**0.52**	0.54	0.45	N											
LP	**−**0.22	**−**0.30	**−0.27**	0.27	**−**0.23	LP										
LBF	0.21	**0.79**	**0.76**	**−**0.14	0.09	0.08	LBF									
LF	**−**0.10	**0.77**	**0.76**	0.05	0.24	**−**0.16	**0.82**	LF								
LHF	0.22	0.002	0.03	0.47	**−**0.40	**0.51**	0.25	0.19	LHF							
LChl	**−**0.21	0.38	0.33	**−**0.02	0.21	0.21	0.08	0.09	0.16	LChl b						
SP	**−**0.23	**−**0.36	**−**0.34	0.22	**−**0.23	**0.48**	**−**0.15	**−**0.30	0.09	0.04	SP					
SBF	0.35	**−**0.158	**−**0.13	0.39	**−**0.16	0.04	0.05	0.05	0.41	**−**0.23	**−**0.04	SBF				
SF	**0.71**	0.33	0.37	0.37	**−**0.005	0.003	0.39	0.32	0.21	**−**0.11	**−**0.03	**0.71**	sf			
SPr	**−**0.25	**−**0.39	**−**0.36	0.26	**−**0.26	**0.65**	**−**0.11	**−**0.30	0.20	0.09	**0.98**	**−**0.03	**−**0.03	SPr		
SHF	**−**0.09	0.09	0.11	**−**0.20	**0.76**	**−0.46**	**−**0.04	0.07	**−0.48**	**−**0.20	**−**0.40	**−**0.20	**−**0.28	**−**0.45	SHF	
SZea	0.03	**−**0.35	**−**0.33	0.21	**−**0.42	**0.866**	**−**0.02	**−**0.34	0.41	0.23	**0.62**	**−**0.01	0.10	**0.73**	**−0.58**	SZea
SChl b	**−**0.21	**−**0.33	**−**0.34	0.30	**−**0.43	0.42	**−**0.07	**−**0.08	0.38	**0.47**	0.37	0.08	0.19	0.41	**−0.62**	**0.57**

*The significance of the correlation is based on the *p* value (*p* > 0.05) obtained for a 95% confidence interval (data not shown). Values in red are significantly negatively correlated in and values in yellow are significantly positively correlated. The correlation between macronutrients and the pigments representative of the different phytoplankton species found in the community were analyzed. Pigments associated with the large (L, >10 μm) and the small (S, 0.8–10 μm) phytoplankton communities were distinguished. The parameters selected are silicic acid (SI), NO_2_ + NO_3_ (NOx); phosphates (Ph); ammonia (A); nitrite (N); Chlorophyll b (Chl b); Peridin (P); 19-But-Fucoxanthin (BF); Fucoxanthin (F); 19-Hex-fucoxanthin (HF); Prasinoxanthin (Pr); zeaxanthin (Zea)*.

**Table A5:** **Pearson correlation table showing the coefficient of correlation between two parameters considering all stations and al depths (0–125 m)**.

Variables	Co																	
Ni	**0.90**	Ni																
Cu	**0.77**	**0.91**	Cu															
Zn	**0.72**	**0.63**	0.51	Zn														
Cd	**0.90**	**0.93**	**0.82**	**0.75**	Cd													
Fe	**−0.55**	**−0.54**	**−**0.40	**−0.77**	**−0.55**	Fe												
Zn/Co	0.13	0.14	0.08	**0.71**	0.23	**−0.74**	Zn/Co											
LP	**−**0.30	**−**0.23	**−**0.24	**−**0.34	**−**0.22	**0.52**	**−**0.24	LP										
LBF	**0.54**	**0.77**	**0.66**	0.15	**0.62**	**−**0.19	**−**0.28	0.08	LBF									
LF	**0.65**	**0.73**	**0.58**	0.33	**0.64**	**−**0.40	**−**0.18	**−**0.16	**0.82**	LF								
LHF	**−**0.15	**−**0.09	0.08	**−**0.50	**−**0.17	0.47	**−0.67**	**0.51**	0.25	020	LHF							
LChl b	**−**0.08	**−**0.11	**−**0.15	**−**0.05	**−**0.22	0.06	**−**0.04	0.21	0.08	0.09	0.16	LChl b						
SP	**−**0.47	**−**0.27	**−**0.28	**−**0.33	**−**0.30	0.11	0.30	**0.48**	**−**0.15	**−**0.30	0.09	0.04	SP					
SBF	0.18	0.02	0.04	**−**0.20	0.07	0.18	**−**0.37	0.04	0.05	0.05	0.41	**−**0.23	**−**0.04	SBF				
SF	0.34	0.33	0.13	0.16	0.34	**−**0.24	**−**0.02	0.003	0.39	0.32	0.21	**−**0.11	**−**0.03	**0.71**	SF			
SPr	**−**0.47	**−**0.28	**−**0.30	**−**0.37	**−**0.32	0.22	0.21	**0.65**	**−**0.11	**−**0.30	0.20	0.09	**0.98**	**−**0.03	**−**0.03	SPr		
SHF	0.26	0.25	0.35	**0.64**	0.27	0.42	0.43	**0.460**	**−**0.04	0.07	**−0.48**	**−**0.20	**−**0.40	**−**0.20	**−**0.28	**−**0.45	SHF	
SZea	**−0.55**	**−**0.37	**−**0.38	**−**0.54	**−**0.40	**0.55**	**−**0.20	**0.86**	**−**0.02	**−**0.34	0.41	0.23	**0.62**	**−**0.01	0.10	**0.73**	**−0.58**	SZea
SChl b	**−0.48**	**−0.49**	**−0.58**	**−**0.36	**−0.49**	0.21	**−**0.03	0.42	**−**0.07	**−**0.08	0.38	**0.47**	0.37	0.08	0.20	0.41	**−0.62**	**0.57**

*The significance of the correlation is based on the *p* value (*p* > 0.05) obtained for a 95% confidence interval (data not shown). Values in red are significantly negatively correlated in and values in yellow are significantly positively correlated. The correlation between micronutrients and the pigments representative of the different phytoplankton species found in the community were analyzed. Pigments associated with the large (L, >10 μm) and the small (S, 0.8–10 μm) phytoplankton communities were distinguished. The parameters selected are cobalt (Co); nickel (Ni); copper (Cu); zinc (Zn); cadmium (Cd); iron (Fe); sum of Chlorophyll c (Chl c sum); Chlorophyll b (Chl b); Peridin (P); 19- But-Fucoxanthin (BF); Fucoxanthin (F); 19-Hex-fucoxanthin (HF); Prasinoxanthin (Pr); zeaxanthin (Zea). Fe data are from Lannuzel et al. (2011)*.
